# Associations of socioeconomic status indicators and migrant status with risk of a low vegetable and fruit consumption in children

**DOI:** 10.1016/j.ssmph.2022.101039

**Published:** 2022-02-04

**Authors:** Mirte Boelens, Hein Raat, Anne I. Wijtzes, Gea M. Schouten, Dafna A. Windhorst, Wilma Jansen

**Affiliations:** a Department of Public Health, Erasmus MC, University Medical Center Rotterdam, PO Box 2040, 3000, CA, Rotterdam, the Netherlands; b Department of Research and Business Intelligence, Municipality of Rotterdam, PO Box 21323, 3001, AH, Rotterdam, the Netherlands; c Department of Social Development, Municipality of Rotterdam, PO Box 70032, 3000, LP, Rotterdam, the Netherlands

**Keywords:** Vegetables, Fruit, Socioeconomic status, Child, Food consumption, Netherlands, SES, Socioeconomic Status, NSES, Neighbourhood socioeconomic status, OR, Odds ratio, CI, Confidence interval, MOR, Median odds ratio, VIF, Variance inflation factor, IQR, Interquartile range

## Abstract

**Background:**

It is important to provide insight in potential target groups for interventions to reduce socioeconomic inequalities in children's vegetable/fruit consumption. In earlier studies often single indicators of socioeconomic status (SES) or migrant status have been used. However, SES is a multidimensional concept and different indicators may measure different SES dimensions. Our objective is to explore multiple associations of SES indicators and migrant status with risk of a low vegetable/fruit consumption in a large multi-ethnic and socioeconomically diverse sample of children.

**Methods:**

We included 5,010 parents of 4- to 12-year-olds from a Dutch public health survey administered in 2018. Cross-sectional associations of parental education, material deprivation, perceived financial difficulties, neighbourhood socioeconomic status (NSES) and migrant status with low (≤4 days a week) vegetable and fruit consumption in children were assessed using multilevel multivariable logistic regression models. Results are displayed as odds ratios (OR) with 95% confidence intervals (CI).

**Results:**

Of the 4- to 12-year-olds, 22.1% had a low vegetable consumption and 11.9% a low fruit consumption. Low (OR 2.51; 95%CI: 2.05, 3.07) and intermediate (OR 1.83; 95%CI: 1.54, 2.17) parental education, material deprivation (OR 1.45; 95%CI: 1.19, 1.76), low NSES (OR 1.28; 95%CI: 1.04, 1.58) and a non-Western migrant status (OR 1.94; 95%CI: 1.66, 2.26) were associated with a higher risk of a low vegetable consumption. Low (OR 1.68; 95%CI: 1.31, 2.17) and intermediate (OR 1.39; 95%CI: 1.12, 1.72) parental education and material deprivation (OR 1.63; 95%CI: 11.27, 2.08) were also associated with a higher risk of a low fruit consumption.

**Conclusion:**

Our findings indicate associations of multiple SES indicators and migrant status with a higher risk of a low vegetable/fruit consumption in children and thus help to identify potential target groups.

## Highlights

• Vegetable and fruit consumptions on 4 days or less a week is reported for respectively 22.1% and 11.9% of children.
• Children's vegetable but not fruit consumption differs between neighbourhoods.
• A non-Western migrant status and a low socio-economic status of the neighbourhood are associated with low vegetable consumption.
• Low/intermediate education and material deprivation are associated with low vegetable and fruit consumption.
• This study provides clues to potential entry points for reducing inequalities in vegetable and fruit consumption.

## 1 Introduction

The consumption of sufficient vegetables and fruits during childhood is important for growth and development and influences health outcomes in later life (Aune et al., 2017; Spence et al., 2018). Many children worldwide do not meet the recommendations for vegetable and fruit consumption (Yngve et al., 2005). Socially disadvantaged children, especially, are at increased risk of not meeting these recommendations (Rasmussen et al., 2006). Socio-ecological models integrate the intrapersonal/individual, interpersonal, community, and organizational and public policy levels which interact and influence health behaviours (de Villiers & Faber, 2019; Golden & Earp, 2012; McLaren & Hawe, 2005). Public policies can improve dietary behaviour by targeting specific intrapersonal/individual characteristics, such as family socioeconomic status (SES) and neighbourhood socioeconomic status (NSES), (de Villiers & Faber, 2019; Golden & Earp, 2012; McLaren & Hawe, 2005). SES is a multidimensional concept that entails multiple related indicators (Braveman et al., 2005). In the literature on health inequalities, family and neighbourhood indicators on income/poverty and educational level are measures that are often used (Braveman et al., 2005; Rasmussen et al., 2006). Since ethnic minority groups are often disadvantaged groups, migrant status closely relates to SES indicators. Furthermore, migrant status may also be related to dietary behaviour because of cultural differences in food choices and patterns. Different SES indicators may measure different dimensions of SES (Braveman et al., 2005). When aiming to identify possible target groups it is thus important to study associations of multiple SES indicators and migrant status with low vegetable and fruit consumption. Previous research has reported associations of different SES indicators and migrant status with low vegetable and fruit consumption. Higher parental education has been associated with higher vegetable and fruit consumption in parents and their children (Rodenburg et al., 2012). As such, parental education is hypothesized to be associated with parenting practices and knowledge about health benefits of vegetable and fruit consumption (Vereecken & Maes, 2010). Other SES indicators, such as material deprivation and perceived financial difficulties, may indicate the inability of parents to purchase sufficient vegetables and fruit for their children. Studies have demonstrated that children consume less vegetables and fruits if their parents reported difficulties in buying food or reported financial difficulties (Macfarlane et al., 2010; Williams et al., 2012). In multiple countries, energy-dense foods are cheaper than nutrient-dense foods (Kirkpatrick & Tarasuk, 2007). low-income groups often have more energy-dense diets lacking sufficient vegetables and fruits (Kirkpatrick & Tarasuk, 2007). Neighbourhoods with a low NSES may have less healthy food facilities and more unhealthy food facilities (Hobbs et al., 2021). It is hypothesized that this situation may lead to fewer vegetable and fruit purchases by parents (Pinho et al., 2020; Reidpath et al., 2002). Studies from the USA have shown that a child's migrant status may be associated with vegetable and fruit consumption in either direction (Guerrero & Chung, 2016; Satia-Abouta et al., 2002). It is suggested that this could possibly be due to differences in traditions, religion, beliefs, practices, food preferences and availability of preferred foods. Also, acculturation and adoption of the diet of the host country might diminish diet-related cultural differences (Guerrero & Chung, 2016; Satia-Abouta et al., 2002). Integration is complex and depends on many aspects including but not limited to language, education, employment and accommodation. For example, integration could occur more easily in mixed communities (e.g. neighbourhoods) than in ghettos or ethnic enclaves with segregation (Lamanna et al., 2018; Satia-Abouta et al., 2002). This is also found in a study in which integration of Syrian refugees in Turkey was measured using cell phone data (Bakker et al., 2019). The authors reported that in Istanbul, compared to touristic area's and to Anatolia, had as higher integration of Syrian refugees due to more mixed communities and more interaction with local inhabitants (Bakker et al., 2019). Better integration and more between migrants and locals could lead to more acculturation such as adopting the diet of the host country (Satia-Abouta et al., 2002).Unfortunately, earlier studies that have studied associations of SES indicators and migrant status with low vegetable and fruit consumption in children often used a single indicator (Guerrero & Chung, 2016; Rasmussen et al., 2006; Zimmer et al., 2019). As different SES indicators may measure different dimensions of SES it is important to study multiple SES indicators when examining possible target groups. Moreover, studies on migrant status are often from the USA, leading to findings that might not be directly comparable to European children due to differences in migration histories and host countries (Guerrero & Chung, 2016; Satia-Abouta et al., 2002; Zimmer et al., 2019). Hence, we studied associations of multiple SES indicators (parental education, material deprivation, perceived financial difficulties, NSES) and migrant status with low vegetable and fruit consumption in a large, socioeconomically and ethnically diverse population-based sample of 4- to 12-year-olds living in the Netherlands.

## 2 Methods

### 2.1 Study setting and participants

Data were obtained from a cross-sectional Dutch Public Health survey carried out in 2018 by the municipal public health service in the city of Rotterdam. A random probability sample of parents of 0- to 12-year-olds living in Rotterdam stratified by neighbourhood was invited to participate. Parents received invitation letters with information and login details for the online survey. Hardcopy questionnaires were available in Dutch, English, or Turkish, and were enclosed with both reminders. The main caregiver was invited to complete the questionnaire. Non-responders were contacted by telephone. If needed, help was offered by clarifying questions so parents were able to complete the questionnaire. Additional effort was made to target parents with Turkish and Moroccan backgrounds and residents of neighbourhoods with a low response. The sample consists of N = 5,010 parents/caregivers of 4- to 12-year-olds. The response rate was 34% and varied between 23% and 54%, depending on the neighbourhood. Response rates did not differ by age or gender of the children. These data were linked to the most recent data about the NSES (2017) provided by the Netherlands Institute of Social Research (SCP). NSES scores were matched to individual questionnaire data using the neighbourhood code (based on postal codes). We compared children with complete data (N = 3,946) to children with one or more missing data (N = 1,064). Children with missing data more often have higher educated parents, no material deprivation, more often lived in neighbourhoods with low NSES and with a Western migrant status (p < 0.01), but did not show difference in age, gender, perceived financial difficulties, or vegetable and fruit consumption (p > 0.05).

### 2.2 Data availability

The data underlying this article are provided by the municipal public health service in the city of Rotterdam and by the SCP. Data will be shared upon request to the corresponding author with the permission of the municipal public health service in the city of Rotterdam and the SCP.

### 2.3 Measures on the family/individual level

#### 2.3.1 Parental education

Parental education was defined as the highest educational level obtained by either one of the parents. The main caregiver filled out the educational level of both parents on the questionnaire. Parental education was categorized as ‘low education’ (i.e. no education, primary school, or ≤4 years general secondary school),‘intermediate education’ (i.e. >4 years general secondary school or intermediate vocational training), and ‘higher education’ (i.e. higher vocational training, university degree, or higher) based on the Dutch Standard Classification of Education (Statistics Netherlands, 2017).

#### 2.3.2 Material deprivation

Eight statements assessed material deprivation (i.e. what parents cannot afford due to a lack of money).The statements resemble the EU-SILC (European Union Statistics on Income and Living Conditions) questions (Arora et al., 2015) but are targeted specifically at children: my child cannot:

1) be a member of a sports club,
2) be a member of another club such as theatre or music,
3) attend birthday parties or trips with school,
4) cannot go on holiday or days-out,
5) eat fruit or vegetables daily,
6) attend swimming lessons,
7) visit a care provider if that is actually necessary, and
8) receive the medication or care that is needed.

Answer categories were ‘true’, ‘somewhat true’, and ‘not true’. We dichotomized the answers to ‘yes’ (true and somewhat true) and ‘no’ (not true). The answers to these eight statements resulted in a material deprivation score ranging from zero to eight (eight being the highest score i.e. parents could not afford any of the eight items). Internal consistency was good (Cronbach's alpha of 0.85). Due to a skewed distribution, the scale was dichotomized into ‘no material deprivation’, i.e. parents could afford all eight items, and ‘material deprivation’, i.e. parents could not afford one or more items.

#### 2.3.3 Perceived financial difficulties

Perceived financial difficulties were assessed by the question “Have you had difficulties in the past twelve months making ends meet with your household income?“, with answer categories: ‘no’, ‘no but I do have to keep an eye on what I spend’, ‘yes some difficulty’, and ‘yes a lot of difficulty’. The answers were dichotomized as either: ‘no’ (answer categories ‘no’ and ‘no, but I do have to keep an eye on what I spend’) or ‘yes’ (answer categories ‘yes some difficulty’ and ‘yes a lot of difficulty’).

#### 2.3.4 Migrant status

Migrant status of the child was defined as ‘Western migrant or Dutch’ or ‘non-Western migrant’. A non-Western migrant status was assigned when the child itself or either (or both) of the parents were born in a non-Western country (Statistics Netherlands, 2012).The Following countries were considered Western: Europe (except for Turkey), North America, Oceania, Indonesia and Japan (Statistics Netherlands). People from Indonesia and Japan are considered Western due to their socioeconomic and cultural position (Statistics Netherlands).

### 2.4 Measure on the neighbourhood level

#### 2.4.1 Neighbourhood socioeconomic status

The SCP computed NSES scores using principal component analysis based on registry data from 2017 on mean income, percentage low incomes, percentage low educated residents, and percentage unemployed residents in a neighbourhood (Netherlands Institute of Social Research, 2019). These NSES scores are standardized scores based on all other neighbourhoods in the Netherlands. These data were matched to the questionnaire data using the neighbourhood code (based on postal codes). In total, for 49 of the 57 neighbourhoods in our study NSES could be matched. We dichotomized NSES to either a ‘high’ or a ‘low’ NSES using a median split.

### 2.5 Study outcomes

#### 2.5.1 Vegetable consumption

Children's vegetable consumption was assessed using the following question: On how many days a week does your child eat vegetables? The question had eight answer categories: ‘Almost never’, ‘one day’, ‘two days’, ‘three days’, ‘four days’, ‘five days’, ‘six days’, and ‘every day’. We dichotomized vegetable consumption as ≤4 days a week i.e. ‘low’ versus >4 days a week i.e. ‘higher’. Higher vegetable consumption was used as the reference group.

#### 2.5.2 Fruit consumption

Children's fruit consumption was assessed using the following question: On how many days a week does your child eat fruit? The question had eight answer categories: ‘Almost never’, ‘one day’, ‘two days’, ‘three days’, ‘four days’, ‘five days’, ‘six days’, and ‘every day’. We dichotomized fruit consumption as ≤4 days a week i.e. ‘low’ versus >4 days week i.e. ‘higher’. Higher fruit consumption was used as the reference group.

#### 2.5.3 Confounders

Age, gender, and family situation of the child were considered confounders and derived from the public health survey (Braveman et al., 2005; Rasmussen et al., 2006). Age was measured in years. Gender was measured dichotomously with ‘boys’ as the reference group. Family situation was dichotomized as either ‘two-parent family’ or ‘single-parent/other family situation’ with ‘two-parent family’ as the reference group.

#### 2.5.4 Statistical analyses

**Fig. 1:**
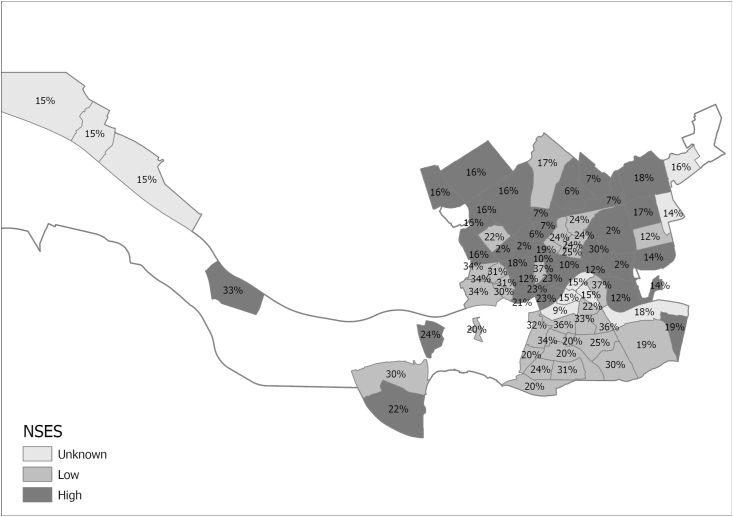
Distribution of a low vegetable consumption of children across neighbourhoods with a low or high neighbourhood socioeconomic status in Rotterdam in the Netherlands.A low vegetable consumption was a vegetable consumption on ≤4 days per week. NSES = neighbourhood socioeconomic status.

**Fig. 2:**
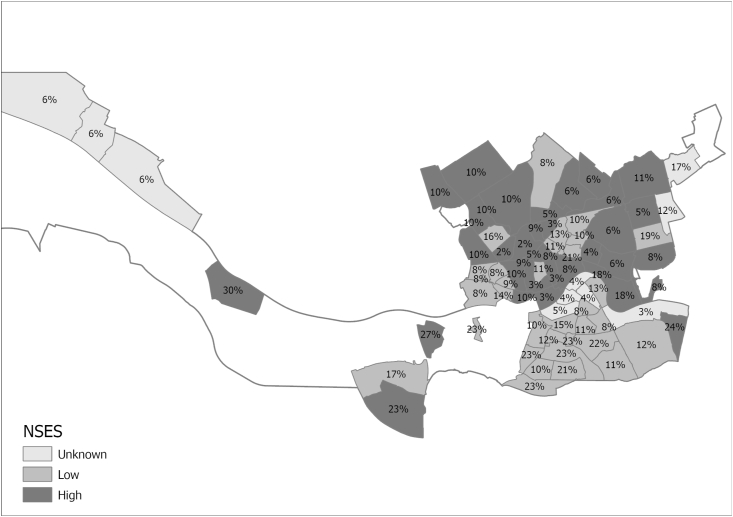
Distribution of a low fruit consumption of children across neighbourhoods with a low or high neighbourhood socioeconomic status in Rotterdam in the Netherlands.A low fruit consumption was a fruit consumption on ≤4 days per week. NSES = neighbourhood socioeconomic status.

Normality of the data was inspected for the continuous variable age using the Kolmogorov-Smirnov test and was found to be not normally distributed (p < 0.001). Descriptive statistics (i.e. percentages for categorical variables and median with interquartile range (IQR) for age), chi-square and Mann-Whitney U tests were used to describe and compare children with a low or higher vegetable and fruit consumption. Further, we computed the percentage children with a low vegetable and a low fruit intake in each low and high SES neighbourhood and presented this information on a map for (Fig. 1 and Fig. 2) for a visual inspection of the distribution.

Missing data (ranging between 0.2% and 15.5%, see Table 1.) were imputed in SPSS using a fully conditional specified model based on the relationships between all the variables included in this study (M = 10 datasets). Statistical analyses were performed on each of the imputed datasets and results were pooled using Rubin's Rules.

**Table 1:** Characteristics of the study population, total sample and subsamples according to vegetable and fruit consumption.

	Total sample	Vegetable consumption^1^		P-value	Fruit consumption^2^		P-value
		Higher (>4 days a week)	Low (≤4 days a week)		Higher (>4 days a week)	Low (≤4 days a week)	
**N, (%)**	5,010	3,881 (77.9)	1,099 (22.1)		4,405 (88.1)	595 (11.9)	
**Age, median (IQR)**	8.0 (6.0–10.0)	8.0 (6.0–10.0)	8.0 (6.0–10.0)	0.336	7.0 (6.0–9.0)	8.0 (7.0–10.0)	<0.001
G**ender, % (n)**				0.012			0.001
Girl	48.4 (2,426)	79.5% (1,915)	20.5 (495)		89.6 (2,167)	10.4 (251)	
Boy	51.6 (2,584)	76.5% (1,966)	23.5 (604)		86.7 (2,238)	13.3 (344)	
**Family situation**^**3**^**, % (n)**				<0.001			<0.001
Single or other	25.3 (1,256)	74.1 (924)	25.9 (323)		85.1 (1,066)	14.9 (187)	
Two-parent	74.7 (3,717)	79.4 (2,936)	20.6 (761)		89.2 (3,308)	10.8 (402)	
**Parental education**^**4**^**, % (n)**				<0.001			<0.001
Low	16.6 (796)	62.9 (498)	37.1 (294)		82.5 (655)	17.5 (139)	
Intermediate	32.3 (1,554)	72.3 (1,112)	27.7 (425)		85.8 (1,330)	14.2 (221)	
Higher	51.1 (2,459)	87.4 (3,751)	12.6 (310)		91.7 (2,251)	8.3 (204)	
**Material deprivation**^**5**^**, % (n)**				<0.001			<0.001
Yes	22.1 (1,086)	66.8 (721)	33.2 (359)		82.7 (896)	17.3 (187)	
No	77.9 (3,828)	81.5 (3,099)	18.5 (705)		89.6 (3,425)	10.4 (396)	
**Perceived financial difficulties**^**6**^**, % (n)**				<0.001			<0.001
Yes	15.3 (756)	66.9 (504)	33.1 (249)		84.2 (635)	15.8 (119)	
No	84.7 (4,197)	80.0 (3,337)	20.0 (833)		88.8 (3,719)	11.2 (470)	
**NSES**^**7**^**, % (n)**				<0.001			0.880
Low	55.0 (2,328)	71.4 (1,652)	28.6 (661)		87.6 (2,036)	12.4 (288)	
High	45.0 (1,907)	83.2 (1,578)	16.8 (319)		87.5 (1,666)	12.5 (239)	
**Migrant status**^**8**^**, % (n)**				<0.001			0.006
Non-Western	41.1 (2,046)	68.0 (1,380)	32.0 (648)		86.6 (1,767)	13.4 (274)	
Western	58.9 (2,938)	84.9 (2,485)	15.1 (441)		89.1 (2,614)	10.9 (319)	

Study population consists of 4- to 12-year-olds (N = 5,010) measured by a public health survey in 2018, the Netherlands. Percentages are row percentages for the stratified analyses and column for the total population. Statistical significance tested by Chi-square for categorical data and by Mann-Whitney U tests for continuous data. NSES=Neighbourhood Socioeconomic Status; ^1^ 30 are missing (0.6%); ^2^ 10 are missing (0.2%); ^3^ 37 are missing (0.7%); ^4^ 201 are missing (4%); ^5^ 96 are missing (1.9%); ^6^ 57 are missing (1.1%); ^7^775 are missing (15.5%); ^8^26 are missing (0.5%).

Associations of parental education, material deprivation, perceived financial difficulties, NSES and migrant status with low vegetable and fruit consumption in children were assessed using multilevel multivariable logistic regression analyses. A random intercept for neighbourhood and fixed slopes model was used to obtain the odds ratio (OR) and corresponding 95% confidence interval (CI) of the risk of low vegetable and fruit consumption. First, an intercept-only model was computed to obtain the median odds ratio (MOR). The MOR quantifies the magnitude of the variation in vegetable and fruit consumption that is explained by the neighbourhood level and varies between one (no variation) and infinity. Subsequently, three models were computed, i.e. crude models (unadjusted models containing one SES indicator or migrant status only), confounder adjusted models, and full models adjusting for confounders and all other SES indicators and migrant status.

Interaction effects of sociodemographic variables (age, sex, and family situation) with SES indicators and migrant status were assessed by adding interaction terms one by one in the fully adjusted models. In a similar vein, interaction effects were investigated between all indicators of SES and migrant status. Bonferroni correction was applied for multiple testing when investigating the interaction effects (p = 0.05/25 = 0.002). Multicollinearity was examined using Spearman's rho coefficients (all <0.7) and VIF values (all<3). Sensitivity analyses using the complete-case sample (N = 3,946) and using non-daily vegetable and fruit consumption as outcome variables were performed. All p-values were two-tailed and level of significance was set at 0.05. Statistical analyses were performed using IBM SPSS statistics for Windows, version 25.0 (International Business Machines Corporation, Armonk, New York).

## 3 Results

### 3.1 General results

Table 1 presents characteristics of our study population. Daily vegetable and fruit consumption was reported for 46.5% and 65.5% of children, while a low consumption (≤4 days a week) was reported for 22.1% and 11.9%. Median age was 8.0 years (IQR = 6.0–10.0) and 48.4% were girls. The children who more often had a low vegetable and fruit consumption were boys or children from single-parent families (or other non-two parent families), from families with lower educated parents, from families with parents who experienced material deprivation or from families with parents who perceived financial difficulties (p < 0.05). Furthermore, children from neighbourhoods with a low NSES more often had a low vegetable consumption (p < 0.05). Fig. 1, Fig. 2 show the distribution of low and high SES neighbourhoods and the corresponding percentage of children with a low vegetable and fruit consumption. The percentage of children with a low vegetable consumption ranged from 2% to 27% depending on the neighbourhood. For fruit the percentage of children with a low consumption ranged between 2% and 30%. Neighbourhoods with a low NSES were often in close proximity of other neighbourhoods with a low NSES and vice versa.

### 3.2 Vegetable consumption

Table 2 shows the results of the regression analyses for a low vegetable consumption. The MOR for vegetable consumption in children in the intercept-only model is 1.66, indicating neighbourhood variance in vegetable consumption. Low (OR 2.51; 95% 2.05, 3.07) and intermediate parental education (OR 1.83; 95%CI: 1.54, 2.17), material deprivation (OR 1.45; 95%CI: 1.19, 1.76) a low NSES (OR 1.28 95%CI: 1.04, 1.58) and a non-Western migrant status (OR 1.94; 95%CI: 1.66, 2.26) were associated with low vegetable consumption. Perceived financial difficulties were not associated with low vegetable consumption.

**Table 2:** Associations of SES indicators and migrant status with low vegetable consumption in N = 5,010 4- to 12-year-olds.

	Null model OR (95% CI)	Model 1 OR (95% CI)	Model 2 OR (95% CI)	Model 3 OR (95% CI)
		**Separate models**		**Combined model**
**Parental education**				
Low		**3.18 (2.62, 3.84)**	**3.17 (2.61, 3.86)**	**2.51 (2.05, 3.07)**
Intermediate		**2.15 (1.82, 2.53)**	**2.15 (1.82, 2.54)**	**1.83 (1.54, 2.17)**
Higher		Ref	Ref	Ref
**Material deprivation**				
Yes		**1.97 (1.69, 2.30)**	**1.96 (1.68, 2.29)**	**1.45 (1.19, 1.76)**
No		Ref	Ref	Ref
**Perceived financial difficulties**				
Yes		**1.75 (1.48, 2.08)**	**1.72 (1.44, 2.06)**	1.16 (0.93, 1.45)
No		Ref	Ref	Ref
**NSES**				
Low		**1.87 (1.48, 2.35)**	**1.84 (1.46, 2.31)**	**1.28 (1.04, 1.58)**
High		Ref	Ref	Ref
**Migrant status**				
Non-Western		**2.46 (2.12, 2.85)**	**2.44 (2.10, 2.83)**	**1.94 (1.66, 2.26)**
Western		Ref	Ref	Ref
**MOR**	1.66			1.31

Study population consists of 4- to 12-year-olds (N = 5,010) measured by a public health survey in 2018, the Netherlands. Low vegetable consumption indicates a consumption on ≤4 days a week. SES=Socioeconomic Status; OR=Odds Ratio; CI=Confidence interval; NSES=Neighbourhood Socioeconomic Status; MOR=Median Odds Ratio (exp(sqrt(2*variance random intercept)*0.6745)); Numbers in **bold** indicate significance (P < 0.05) Null model=intercept only; model 1 is a crude, unadjusted model; model 2 is adjusted for the age, gender (boy=ref) and family situation of the child (two-parent family=ref); model 3 is model 2 and additionally adjusted for all indicators of socioeconomic status and migrant status.

### 3.3 Fruit consumption

Table 3 shows the results of the regression analyses for low fruit consumption. The MOR in the intercept-only model for fruit consumption in children is 1.58, indicating neighbourhood variance in fruit consumption. Low (OR 1.68; 95%CI: 1.31, 2.17) and intermediate parental education (OR 1.39; 95%CI: 1.12, 1.72) and material deprivation (OR 1.63; 95%CI: 1.27, 2.08) were associated with low fruit consumption. Perceived financial difficulties, NSES and migrant status were not associated with low fruit consumption.

**Table 3:** Associations of SES indicators and migrant status with low fruit consumption in N = 5,010 4- to 12-year-olds.

Parental education	Null model OR (95% CI)	Model 1 OR (95% CI)	Model 2 OR (95% CI)	Model 3 OR (95% CI)
		Separate models		Combined model
Low		**2.05 (1.62, 2.60)**	**1.84 (1.44, 2.34)**	**1.68 (1.31, 2.17)**
Intermediate		**1.55 (1.26, 1.90)**	**1.49 (1.21, 1.83)**	**1.39 (1.12, 1.72)**
Higher		Ref	Ref	Ref
**Material deprivation**				
Yes		**1.75 (1.45, 2.12)**	**1.69 (1.39, 2.06)**	**1.63 (1.27, 2.08)**
No		Ref	Ref	Ref
**Perceived financial difficulties**				
Yes		**1.41 (1.13, 1.76)**	**1.33 (1.06, 1.67)**	0.90 (0.68, 1.19)
No		Ref	Ref	Ref
**NSES**				
Low		1.19 (0.90, 1.58)	1.15 (0.86, 1.53)	0.96 (0.72, 1.28)
High		Ref	Ref	Ref
M**igrant status**				
Non-Western		**1.36 (1.13, 1.64)**	**1.29 (1.07, 1.56)**	1.11 (0.91, 1.35)
Western		Ref	Ref	Ref
**MOR**	1.58			1.54

Study population consists of 4- to 12-year-olds (N = 5,010) measured by a public health survey in 2018, the Netherlands. Low fruit consumption indicates a consumption on ≤4 days a week. SES=Socioeconomic Status; OR=Odds Ratio; CI=Confidence interval; NSES=Neighbourhood Socioeconomic Status; MOR=Median Odds Ratio (exp(sqrt(2*variance random intercept)*0.6745)); Numbers in **bold** indicate significance (P < 0.05) Null model=intercept only; model 1 is a crude, unadjusted model; model 2 is adjusted for age, gender (boy=ref) and family situation of the child (two-parent family=ref); model 3 is model 2 and additionally adjusted for all indicators of socioeconomic status and migrant status.

### 3.4 Additional analyses

We found no significant interaction effects (Supplemental Table 1).

### 3.5 Sensitivity analyses

Sensitivity analyses using non-daily vegetable and fruit consumption as outcome variables were similar but NSES and migrant status were not associated with vegetable consumption and lower parental education was not associated with fruit consumption (Supplemental Tables 2 and 3). Sensitivity analyses using a complete-case sample yielded similar results (Supplemental Tables 4 and 5).

## 4 Discussion

In this large socioeconomically and ethnically diverse population-based sample of 4- to 12-year-olds, we observed that having low/intermediate educated parents, parents who experience material deprivation, being from a low NSES neighbourhood and having a non-Western migrant status is associated with a higher risk of a low vegetable consumption. Furthermore, having low/intermediate educated parents or having parents who experience material deprivation is associated with a higher risk of a low fruit consumption.

We found that, in our sample, 22.1% had a low vegetable consumption. Of all children, 11.9% had a low fruit consumption. The low vegetable and fruit consumption is comparable to findings in other studies among European children (Spence et al., 2018; National Institute for Public Health and the Environment (RIVM), 2016; Lynch et al., 2014).

We observed associations of low/intermediate parental education with low vegetable and fruit consumption in children. This is in line with previous research among European 4- to 11-year-olds (Fernandez-Alvira et al., 2015). In previous research it has been observed that parental vegetable/fruit consumption, self‐efficacy, attitudes, preferences, knowledge, and intentions mediated the association of parental education with children's vegetable and fruit consumption (van Ansem et al., 2014; Zarnowiecki et al., 2014). These factors may explain the association of parental education with children's vegetable and fruit consumption.

We furthermore observed associations of material deprivation with low vegetable and fruit consumption. This is in agreement with a Canadian study in which 3,099 1- to 5-year-olds from parents reporting difficulty buying food had higher odds of consuming less vegetables and fruits (Fuller et al., 2017). Furthermore, in several American studies it has been observed that costs of vegetables and fruits limited their availability at home and were a barrier for adequate vegetable and fruit consumption (Ard et al., 2007; Mushi-Brunt et al., 2007). Adding to this, in the Netherlands, higher dietary costs were associated with healthier foods (Statistics Netherlands, 2007). Moreover, the prices of healthy foods increased more than prices of unhealthy foods in the past years (Statistics Netherlands, 2007). Interestingly, we found no association of perceived financial difficulties with low vegetable or fruit consumption. It could be that some parents who reported material deprivation perceived no financial difficulties or vice versa (Bradshaw & Finch, 2003). Indeed, 18.2% reported financial difficulties and no material deprivation and 11.2% reported material deprivation and no financial difficulties.

In our study we observed an association of living in a neighbourhood with a low NSES with a low vegetable consumption but not a low fruit consumption in children. This partially support findings from earlier studies such as the Young Finns Cohort study in which the prevalence of a low vegetable and fruit consumption was higher in children from neighbourhoods with a low NSES (Kivimäki et al., 2018). Earlier studies have reported that there are more convenience stores and fast food outlets in low NSES neighbourhoods (Bivoltsis et al., 2020; Maguire et al., 2015; Timmermans et al., 2018). It is hypothesized that this possibly results in a higher consumption of ready-to-eat foods and a lower consumption of vegetables and fruits (Bivoltsis et al., 2020; Maguire et al., 2015; Timmermans et al., 2018). An effect study on free provision of vegetables and fruits at Dutch primary schools showed a long-term significant increase in fruit consumption but not in vegetable consumption (Tak et al., 2009).The authors suggest that their finding may be due to Dutch eating habits, namely consuming vegetables at home during dinner whereas fruits are mainly consumed during the day at school (Tak et al., 2009). One could postulate that, because of school policies, NSES has less influence on fruit consumption. At the time of data collection, there were school prevention programs with fruit components implemented in Rotterdam, but data on the reach of these programs is missing. The impact on our results is therefore unclear.

We also observed that a non-Western migrant status was associated with a low vegetable consumption but not with a low fruit consumption. As previously mentioned, fruit consumption is possibly more influenced by school policies than vegetable consumption (Tak et al., 2009). According to a systematic mapping review, differences in beliefs and perceptions of healthy foods, acculturation, and socialization may play a role in dietary behaviours in ethnic minorities living in Europe (Osei-Kwasi et al., 2016). Our results differ from earlier research in which non-Western children consumed more vegetables and fruits than Western children (Osei-Kwasi et al., 2016). However, these children in earlier research mostly were adolescents or data were analyzed together with adults. Also, these studies took place in other European countries than our study (i.e. Norway, Denmark, Switzerland, Croatia and most from the United Kingdom). Some of these countries such as the United Kingdom have a different composition and origin of migrants and migration than the Netherlands (Office for National Statistics). To illustrate, in the Netherlands most non-Western migrants have a Turkish, Moroccan or Surinamese background and in the United Kingdom most non-Western migrants have an Indian, Pakistani, Chinese or Bangladeshi background (Office for National Statistics; Statistics Netherlandsb). Also, these studies were performed between 2000 and 2011 (Osei-Kwasi et al., 2016). The children with a migrant status in our sample were mostly born in the Netherlands and were thus second generation migrant children (82.6%). It is hypothesized that these children and their parents could have experienced more acculturation towards a Western diet. This is in concordance with a systematic review that studied acculturation in relation to weight gain in high-income countries. In this review an overall positive association of a higher degree of acculturation of migrants with obesity was found (Delavari et al., 2013). We categorized children from non-Western countries in one group and children from Western countries in another group. Non-Western countries or Western countries may differ from each other economy, religion, culture, diet, and lifestyle. Therefore, these groups could be heterogeneous regarding diet and lifestyle.

**Fig. 3:**
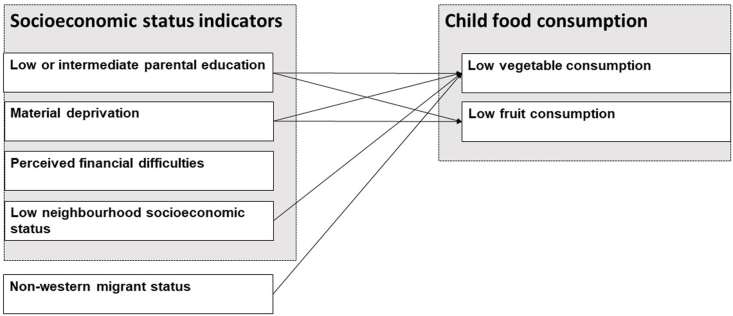
Observed associations of socioeconomic status indicators and migrant status with low vegetable and low fruit consumption in children.

In our study we sought to identify potential target groups at which interventions could be directed. Fig. 3 shows the associations that we found of the SES indicators and migrant status with risk of low vegetable consumption and low fruit consumption in children in our data. We did this by studying associations of multiple SES indicators and migrant status with a low vegetable and fruit consumption jointly. We want to emphasize that our estimates therefore cannot be interpreted as causal effects (Westreich & Greenland, 2013). Likewise, distinguishing direct and indirect effects of our effect estimates is not possible (Westreich & Greenland, 2013). We have not performed causal mediation analysis in our cross-sectional data as this was not the goal of our study. Moreover, using cross-sectional data, no causation or temporal direction can be established. Therefore, we cannot report on possible mediators underlying associations of SES indicators and migrant status with vegetable and fruit consumption. To gain more insight into these associations longitudinal mediation studies are warranted. As we did not perform spatial analyses we do not know whether neighbourhood effects spatially cluster or not. We recommend future research to examine both multilevel and spatial regression jointly to examine both neighbourhood variation as well as the pattern of variation between neighbourhoods (Chaix et al., 2005).

Strengths of this study include the large and diverse population-based sample which are both important for the generalizability of our findings. Another strength is that we could include multiple indicators of socioeconomic status on the family/individual as well as the neighbourhood level into our analysis. There are also some limitations. As previously mentioned, the cross-sectional design of the study makes it impossible to establish causation or a temporal direction. NSES had 15.5% missing values, which could potentially impact the results. However, our complete-case analysis yielded similar results as our multiple-imputed analysis. We used a dichotomized measure of NSES as more categories resulted in empty cells in the multilevel analysis. Also, individual measures used for creating the NSES variable were not available and could not be included in our analysis. We only measured the number of days that children consumed vegetables/fruits. Furthermore, the perception of vegetables/fruits is variable and socially desirable answers could have biased the results (Roark & Niederhauser, 2013). We dichotomized migrant status because of limited participants in some substrata which may have masked subgroup effects. Lastly, residual confounding by unmeasured or imprecisely measured confounders could also be present.

## 5 Conclusion

In conclusion, multiple SES indicators and migrant status are associated with a higher risk of a low vegetable and fruit consumption in 4- to 12-year-olds. Our results are important for researchers, policymakers, and health professionals as they help to identify potential target groups for interventions.

## Funding

This work was funded by a research grant (project number: 531001313) from 10.13039/501100001826ZonMw, 10.13039/100005622The Netherlands Organization for Health Research and Development. ZonMw has no role in any part of the research, writing and reviewing of the manuscript.

## Financial disclosures

None declared.

## CRediT authorship contribution statement

**Mirte Boelens:** Conceptualization, Methodology, Formal analysis, Writing – original draft. **Hein Raat:** Supervision, Conceptualization, Methodology, Writing – review & editing. **Anne I. Wijtzes:** Conceptualization, Writing – review & editing. **Gea M. Schouten:** Data collection, Writing – review & editing. **Dafna A. Windhorst:** Conceptualization, Writing – review & editing. **Wilma Jansen:** Supervision, Overall supervision, Conceptualization, Methodology, Writing – review & editing.

## Declaration of competing interest

None declared.
