# Supplementary material for: Associations of socioeconomic status indicators and migrant status with risk of a low vegetable and fruit consumption in children
Source: SSM Popul Health. 2022 Feb 4;17:101039. doi: 10.1016/j.ssmph.2022.101039 (PMC8841774; doi:10.1016/j.ssmph.2022.101039)
Supplement: Multimedia component 1 [file mmc1.docx]

| **Table S1.** Testing interaction effects in the fully adjusted models. | | |
| --- | --- | --- |
|  | **Low vegetable consumption** | **Low fruit consumption** |
| **Parental education with** |  |  |
| Gender | 0.11 | 0.46 |
| Family situation | 0.59 | 0.59 |
| Age | 0.68 | 0.25 |
| Material deprivation | 0.04 | 0.32 |
| Financial difficulties | 0.61 | 0.27 |
| NSES | 0.01 | 0.18 |
| Migrant status | 0.01 | 0.27 |
|  |  |  |
| **Material deprivation with** |  |  |
| Gender | 0.40 | 0.79 |
| Family situation | 0.56 | 0.92 |
| Age | 0.16 | 0.34 |
| Financial difficulties | 0.19 | 0.12 |
| NSES | 0.27 | 0.25 |
| Migrant status | 0.75 | 0.58 |
|  |  |  |
| **Financial difficulties with** |  |  |
| Gender | 0.75 | 0.35 |
| Family situation | 0.10 | 0.06 |
| Age | 0.10 | 0.71 |
| NSES | 0.97 | 0.46 |
| Migrant status | 0.27 | 0.28 |
|  |  |  |
| **NSES with** |  |  |
| Gender | 0.01 | 0.37 |
| Family situation | 0.70 | 0.43 |
| Age | 0.72 | 0.28 |
| Migrant status | 0.53 | 0.22 |
|  |  |  |
| **Migrant status with** |  |  |
| Gender | 0.42 | 0.14 |
| Family situation | 0.68 | 0.10 |
| Age | 0.20 | 0.18 |
| Interaction effects of sociodemographic variables (i.e. age, gender and family situation), SES indicators and migrant status were assessed by adding interaction terms one by one in the fully adjusted models (model 3). A Bonferroni correction was used to investigate significant interaction effects (p=0.05/25=0.002) NSES=Neighborhood SES). | | |

| **Table S2.** Associations of socioeconomic indicators and migrant status with non-daily vegetable consumption (N=5,010). | | | | |
| --- | --- | --- | --- | --- |
|  | **Null model OR (95% CI)** | **Model 1**  **OR (95% CI)** | **Model 2**  **OR (95% CI)** | **Model 3**  **OR (95% CI)** |
| **Parental education** |  | **Separate models** | | **Combined model** |
| Low |  | **1.88 (1.59, 2.23)** | **1.88 (1.59, 2.24)** | **1.76 (1.47, 2.11)** |
| Intermediate |  | **1.56 (1.36, 1.78)** | **1.56 (1.37, 1.79)** | **1.48 (1.29, 1.70)** |
| Higher |  | Ref | Ref | Ref |
| **Material deprivation** |  |  |  |  |
| Yes |  | **1.48 (1.28, 1.70)** | **1.47 (1.28, 1.70)** | **1.39 (1.16, 1.66)** |
| No |  | Ref | Ref | Ref |
| **Perceived financial difficulties** |  |  |  |  |
| Yes |  | **1.24 (1.06, 1.45)** | **1.23 (1.04, 1.44)** | 0.92 (0.75, 1.12) |
| No |  | Ref | Ref | Ref |
| **NSES** |  |  |  |  |
| Low |  | **1.24 (1.03, 1.49)** | **1.23 (1.02, 1.48)** | 1.07 (0.89, 1.28) |
| High |  | Ref | Ref | Ref |
| **Migrant status** |  |  |  |  |
| Non-Western |  | **1.24 (1.10, 1.40)** | **1.23 (1.09, 1.39)** | 1.06 (0.93, 1.21) |
| Western |  | Ref | Ref | Ref |
| **MOR** | 1.44 |  |  | 1.37 |
| OR=Odds Ratio; CI=Confidence interval; NSES=Neighbourhood Socioeconomic Status; MOR=Median Odds Ratio (exp(sqrt(2*variance random intercept)*0.6745; Numbers in **bold** indicate significance (P <0.05) Null model=intercept only; model 1 is a crude, unadjusted model; model 2 is adjusted for the age, gender (boy=ref) and family situation of the child (two-parent family=ref); model 3 is model 2 and additionally adjusted for all indicators of socioeconomic status and migrant status. | | | | |

| **Table S3.** Associations of socioeconomic indicators and migrant status with non-daily fruit consumption (N=5,010). | | | | |
| --- | --- | --- | --- | --- |
|  | **Null model OR (95% CI)** | **Model 1**  **OR (95% CI)** | **Model 2**  **OR (95% CI)** | **Model 3**  **OR (95% CI)** |
| **Parental education** |  | **Separate models** | | **Combined model** |
| Low |  | **1.28 (1.09, 1.52)** | 1.18 (1.00, 1.40) | 1.17 (0.98, 1.40) |
| Intermediate |  | **1.23 (1.07, 1.40)** | **1.19 (1.03, 1.36)** | **1.17 (1.02, 1.35)** |
| Higher |  | Ref | Ref | Ref |
| **Material deprivation** |  |  |  |  |
| Yes |  | **1.51 (1.31, 1.74)** | **1.49 (1.29, 1.72)** | **1.56 (1.30, 1.87)** |
| No |  | Ref | Ref | Ref |
| **Perceived financial difficulties** |  |  |  |  |
| Yes |  | **1.27 (1.08, 1.49)** | **1.23 (1.05, 1.46)** | 0.92 (0.75, 1.13) |
| No |  | Ref | Ref | Ref |
| **NSES** |  |  |  |  |
| Low |  | 0.92 (0.78, 1.10) | 0.90 (0.76, 1.07) | 0.86 (0.72, 1.03) |
| High |  | Ref | Ref | Ref |
| **Migrant status** |  |  |  |  |
| Non-Western |  | 0.98 (0.86, 1.11) | 0.94 (0.83, 1.07) | 0.87 (0.76, 1.00) |
| Western |  | Ref | Ref | Ref |
| **MOR** | 1.32 |  |  | 1.28 |
| OR=Odds Ratio; CI=Confidence interval; NSES=Neighbourhood Socioeconomic Status; MOR=Median Odds Ratio (exp(sqrt(2*variance random intercept)*0.6745)); Numbers in **bold** indicate significance (P <0.05) Null model=intercept only; model 1 is a crude, unadjusted model; model 2 is adjusted for age , gender (boy=ref) and family situation of the child (two-parent family=ref); model 3 is model 2 and additionally adjusted for all indicators of socioeconomic status and migrant status. | | | | |

| **Table S4.** Complete-case associations of socioeconomic status indicators and migrant status with low vegetable consumption (N=3,946). | | | | |
| --- | --- | --- | --- | --- |
|  | **Null model** | **Model 1**  **OR (95% CI)** | **Model 2**  **OR (95% CI)** | **Model 3**  **OR (95% CI)** |
| **Parental education** |  | **Separate models** | | **Combined model** |
| Low |  | **3,97 (3.21, 4.91)** | **4.00 (3.22, 4.97)** | **3.01 (2.41, 3.77)** |
| Intermediate |  | **2.51 (2.08, 3.03)** | **2.52 (2.08, 3.05)** | **2.07 (1.70, 2.51)** |
| Higher |  | Ref | Ref | Ref |
| **Material deprivation** |  |  |  |  |
| Yes |  | **2.16 (1.82, 2.56)** | **2.15 (1.81, 2.56)** | **1.58 (1.27, 1.97)** |
| No |  | Ref | Ref | Ref |
| **Perceived financial difficulties** |  |  |  |  |
| Yes |  | **1.85 (1.52, 2.24)** | **1.82 (1.49, 2.21)** | 1.13 (0.89, 1.45) |
| No |  | Ref | Ref | Ref |
| **NSES** |  |  |  |  |
| Low |  | **2.19 (1.64, 2.92)** | **2.15 (1.62, 2.87)** | **1.32 (1.01, 1.71)** |
| High |  | Ref | Ref | Ref |
| **Migrant status** |  |  |  |  |
| Non-Western |  | **2.58 (2.19, 3.05)** | **2.57 (2,17, 3.04)** | **2.01 (1.69, 2.39)** |
| Western |  | Ref | Ref | Ref |
| **MOR** | 1.71 |  |  | 1.36 |
| Low vegetable consumption indicates a consumption on ≤4 days a week. OR=Odds Ratio; CI=Confidence interval; NSES=Neighbourhood Socioeconomic Status; MOR=Median Odds Ratio (exp(sqrt(2*variance random intercept)*0.6745)); Numbers in **bold** indicate significance (P <0.05) Null model=intercept only; model 1 is a crude, unadjusted model; model 2 is adjusted for age, gender (boy=ref) and family situation of the child (two-parent family=ref); model 3 is model 2 and additionally adjusted for all indicators of socioeconomic status and migrant status. | | | | |

| **Table S5.** Complete-case associations of socioeconomic status indicators and migrant status with low fruit consumption (N=3,946). | | | | |
| --- | --- | --- | --- | --- |
|  | **Null model OR (95% CI)** | **Model 1**  **OR (95% CI)** | **Model 2**  **OR (95% CI)** | **Model 3**  **OR (95% CI)** |
| **Parental education** |  | **Separate models** | | **Combined model** |
| Low |  | **2.21 (1.70, 2.88)** | **1.98 (1.51, 2.58)** | **1.82 (1.37, 2.40)** |
| Intermediate |  | **1.56 (1.23, 1.97)** | **1.50 (1.18, 1.90)** | **1.40 (1.10, 1.79)** |
| Higher |  | Ref | Ref | Ref |
| **Material deprivation** |  |  |  |  |
| Yes |  | **1.74 (1.40, 2.15)** | **1.64 (1.32, 2.05)** | **1.67 (1.27, 2.19)** |
| No |  | Ref | Ref | Ref |
| **Perceived financial difficulties** |  |  |  |  |
| Yes |  | **1.31 (1.02, 1.69)** | 1.21 (0.93, 1.56) | 0.81 (0.59, 1.10) |
| No |  | Ref | Ref | Ref |
| **NSES** |  |  |  |  |
| Low |  | 1.33 (0.93, 1.90) | 1.27 (0.89, 1.81) | 1.05 (0.74, 1.49) |
| High |  | Ref | Ref | Ref |
| **Migrant status** |  |  |  |  |
| Non-Western |  | **1.27 (1.03, 1.57)** | 1.21 (0.98, 1.49) | 1.03 (0.83, 1.28) |
| Western |  | Ref | Ref | Ref |
| **MOR** | 1.60 |  |  | 1.56 |
| Low fruit consumption indicates a consumption on ≤4 days a week. OR=Odds Ratio; CI=Confidence interval; NSES=Neighbourhood Socioeconomic Status; MOR=Median Odds Ratio (exp(sqrt(2*variance random intercept)*0.6745)); Numbers in **bold** indicate significance (P <0.05) Null model=intercept only; model 1 is a crude, unadjusted model; model 2 is adjusted for age, gender (boy=ref) and family situation of the child (two-parent family=ref); model 3 is model 2 and additionally adjusted for all indicators of socioeconomic status and migrant status. | | | | |
